# Functional Brain Connectivity of Language Functions in Children Revealed by EEG and MEG: A Systematic Review

**DOI:** 10.3389/fnhum.2020.00062

**Published:** 2020-03-12

**Authors:** Isabelle Gaudet, Alejandra Hüsser, Phetsamone Vannasing, Anne Gallagher

**Affiliations:** ^1^Laboratoire d'imagerie optique en neurodéveloppement (LIONLAB), Sainte-Justine University Hospital Research Center, Montréal, QC, Canada; ^2^Department of Psychology, Université de Montréal, Montréal, QC, Canada

**Keywords:** functional connectivity, cerebral networks, language, language development, children, EEG, MEG, connectivity analysis

## Abstract

The development of language functions is of great interest to neuroscientists, as these functions are among the fundamental capacities of human cognition. For many years, researchers aimed at identifying cerebral correlates of language abilities. More recently, the development of new data analysis tools has generated a shift toward the investigation of complex cerebral networks. In 2015, Weiss-Croft and Baldeweg published a very interesting systematic review on the development of functional language networks, explored through the use of functional magnetic resonance imaging (fMRI). Compared to fMRI and because of their excellent temporal resolution, magnetoencephalography (MEG) and electroencephalography (EEG) provide different and important information on brain activity. Both therefore constitute crucial neuroimaging techniques for the investigation of the maturation of functional language brain networks. The main objective of this systematic review is to provide a state of knowledge on the investigation of language-related cerebral networks in children, through the use of EEG and MEG, as well as a detailed portrait of relevant MEG and EEG data analysis methods used in that specific research context. To do so, we have summarized the results and systematically compared the methodological approach of 24 peer-reviewed EEG or MEG scientific studies that included healthy children and children with or at high risk of language disabilities, from birth up to 18 years of age. All included studies employed functional and effective connectivity measures, such as coherence, phase locking value, and Phase Slope Index, and did so using different experimental paradigms (e.g., at rest or during language-related tasks). This review will provide more insight into the use of EEG and MEG for the study of language networks in children, contribute to the current state of knowledge on the developmental path of functional connectivity in language networks during childhood and adolescence, and finally allow future studies to choose the most appropriate type of connectivity analysis.

## Introduction

Language is a highly complex function that is importantly involved in the development of human cognition and social functions (Berwick et al., 2013). With major advances in neuroimaging techniques, the language neural architecture has been increasingly studied in the past 20 years. While several brain regions have been identified as key areas for expressive and receptive language, it is now also widely recognized that the latter relies more on complex neural networks, requiring coordination between distinct neuronal populations and less on independent and specific brain areas (Ardila et al., 2015; Tremblay and Dick, 2016).

Over the past decades, functional brain connectivity (FC) has progressively captured the interest of scientists and clinical researchers working in the field of cognitive neuroscience, leading to the publication of numerous articles on the subject. On a general note, functional connectivity is defined as the statistical relationships between cerebral signals over time and thus potentially allows conclusions to be made regarding the functional interactions between two or more brain regions. Effective connectivity, on the other hand, goes beyond the correlations between cerebral activity and aims at specifying causal relationships through the use of experimental paradigms or models. This allows for an interpretation of the direction of interactions between different cerebral regions (Friston, 2011). With the sharp increase of studies on brain connectivity, researchers have developed and applied increasingly sophisticated analytic strategies that highlight functional or effective connectivity (EC) and that allow a more advanced exploration of interactions between regional structures and networks involved in language development (Bastos and Schoffelen, 2016). In the past few years, novel neuroimaging techniques and methods of analysis have enabled the examination of functional connectivity patterns. Namely, functional magnetic resonance imaging (fMRI) was the neuroimaging technique used in the first published study of brain spontaneous fluctuations, measured at rest (Biswal et al., 1995). Functional magnetic resonance imaging is widely used in brain connectivity studies, mostly due to its high spatial resolution (in millimeters). However, because it relies on the coupling between cerebral blood flow (hemodynamic response) and the underlying neuronal activation, this technique provides only an indirect measure of brain activity. Moreover, even though neuronal events occur within milliseconds, the induced blood-oxygenation changes spread out over several seconds, thereby severely limiting fMRI's temporal resolution (~2–3 s). Techniques such as electroencephalography (EEG) and magnetoencephalography (MEG), on the other hand, provide direct information on neuronal electrical activity and offer higher temporal resolution (<1 millisecond). This is particularly relevant for the study of language functions, because auditory processing and language processing occur within a short time interval of milliseconds (Skeide and Friederici, 2016).

So far, neuronal accounts of language system development largely rely on EEG data (Skeide and Friederici, 2016). Traditionally, electrophysiological data have been examined for event-related potential (ERP), a method that reflects the brain's activity in response to a particular stimulus event. As of now, several metrics can be used to estimate functional connectivity between electrodes.

In order to perform functional connectivity analysis, MEG and EEG (M/EEG) data are commonly transformed into the frequency domain. Measures are thus typically classified by five fundamental frequency bands, mostly defined by their spectral boundaries: delta (<4 Hz), theta (4–7 Hz), alpha (8–12 Hz), beta (13–30 Hz), and gamma (>30 Hz) (Cacioppo et al., 2007), each of which has different functional characteristics and cortical topography (Herrmann et al., 2016). Despite the fact that the definitions of these bands may vary between studies, and the boundaries used in studies of early childhood may be lower (Saby and Marshall, 2012), the interpretation arising from the present systematic review is based on the above definition by Cacioppo et al. (2007).

What is more, development and maturation affect the frequency and synchronization of neural oscillations, both at rest and during a cognitive task. Globally, analyses of resting state networks reveal that slow-wave activity (delta and theta) tends to decrease throughout childhood and adolescence, whereas oscillations in higher frequency (alpha, beta, and gamma) show an increase with age (Uhlhaas et al., 2010). Moreover, FC in childhood is dominated by short-distance local links, which are gradually replaced by long-distance functional connections in adulthood, thus forming mature cerebral networks (Vértes and Bullmore, 2015; Meng and Xiang, 2016; Oldham and Fornito, 2018). The task-related developmental trajectory of neural oscillations is, however, less clear and varies widely depending on the nature of the task.

When it comes to the functional meaning of different frequency bands, previous studies have suggested that brain signals of each frequency band play a different role. First, the coherence of local neuronal populations and bottom-up processing are associated with high-frequency oscillations (Buzsáki et al., 2013; Friederici and Singer, 2015). Slower frequency ranges, on the other hand, are understood to represent the cooperative activity of large-scale neuronal networks and mediate top-down feedback information (Palva and Palva, 2018).

Regarding language processing, the use of FC in the spectral domain is certainly important, but little is known about the association between frequency bands and language networks. Nevertheless, distinctions have been made regarding language processing and frequency band using spectral power analyses. It is argued that different stages of auditory and speech processing, language comprehension, and active speech itself do not rely on the same frequency bands (for an exhaustive review see Kösem and Van Wassenhove, 2017; Meyer, 2018). More specifically, delta range (<4 Hz) has been associated with intonational processing and syntactic comprehension (Kösem and Van Wassenhove, 2017; Meyer, 2018). It plays a role in top-down processing and seems to contribute to the organization of the cortical speech system, which regulates auditory-cortical excitability. It is further implicated in language comprehension, more precisely in the grouping of words into syntactic phrases (Meyer, 2018). It has been pointed out that theta (4–7 Hz) synchronizes with syllabic rates (Giraud and Poeppel, 2012; Meyer, 2018) and that theta coherence increases in tasks involving verbal information retrieval and verbal working memory (Friederici and Singer, 2015; Meyer, 2018). Alpha (8–12 Hz) oscillations may also play a role in verbal working memory (Friederici and Singer, 2015; Meyer, 2018). Beta activity (13–30 Hz) in language processing has been associated with semantic predictions (top-down mechanisms), as well as in syntactic and semantic binding mechanisms. It has also been correlated with verbal memory processes and language production (Weiss and Mueller, 2012). Finally, the gamma band (>30 Hz) has been associated with phonological perception and assessment of the contextual semantic fit of incoming words [bottom-up; (Meyer, 2018)]. The association of functional connectivity based on frequency bands and the different stages of language processing are still subject to investigation.

Several techniques have been proposed in order to measure cerebral activity, thus allowing for the interpretation of brain connectivity. Even though a large range of FC metrics is available in the current literature, the present article is limited to those brain connectivity approaches used in pediatric electrophysiological language research. Thus, FC analysis will not be addressed exhaustively. Only the most commonly used metrics to quantify brain connectivity, such as coherence, phase locking value (PLV), Phase Lag Index (PLI), correlation, Granger causality, and Graph theory, will be briefly described in this review. Complementary reviews on more detailed mathematical analyses of connectivity methods can be consulted elsewhere (e.g., Kida et al., 2015; Bastos and Schoffelen, 2016).

Connectivity analyses in M/EEG traditionally include the examination for changes in coherence between sources or sensors. Coherence can be defined as the covariation in amplitude and phase between two signals and quantifies the linear correlation between two time series, and this on the frequency domain (Bowyer, 2016). It is assumed that the higher the correlation, the more synchronized, and therefore integrated, the signals are. Thus, coherence is sensitive to changes in both power and phase relationships but cannot provide direct information on the true relationship between the two signals (Sakkalis, 2011).

As an alternative to traditional amplitude-based indices of coherence, metrics of phase synchronization have been developed, such as PLV and PLI. Both PLV and PLI compute the consistency of phase difference between two variables over a time period. They provide a measure of the two signals' temporal relationship, independent of their signal amplitude (Lachaux et al., 1999). The PLV approach evaluates the instantaneous phase difference of signals, assuming that the connected areas generate signals whose phases evolve together. Therefore, the phases of the signals are considered synchronous or locked if the difference between them is constant (Bruña et al., 2018). Similarly, PLI estimates the asymmetry of the distribution of phase differences between two signals, but this method is designed to reduce the effect of volume conduction (Stam et al., 2007). The central idea is that a consistent phase difference between two times series (asymmetric distribution, PLI > 0), cannot result from a single source (volume conduction). Overall, phase synchronization metrics are better used for short-duration events such as in event-related studies, to determine the coupling of two signals across trials (Aydore et al., 2013; Bowyer, 2016).

Recently, directed connectivity or EC metrics have been developed to determine the nature of the neural interactions that enable information flux, such as Granger causality in the time domain (Bressler and Seth, 2011) or phase slope index (PSI) in frequency domain (Nolte et al., 2008). Based on phase differences, PSI is a weighted average measure of phase coherency slope between two signals, over a frequency band (Nolte et al., 2008; Bastos and Schoffelen, 2016). Some EC measures rely on the concept of Granger causality, whereby one time series is said to “Granger cause” a second one if the past values of the first improve the prediction of the second. Originally, the concept of Granger causality was applied to time series, but this approach has been extended to the frequency domain (Geweke, 1982), and many multivariate measures can be derived from this model (Sakkalis, 2011).

Similar to fMRI or other neuroimaging techniques, M/EEG data used along with connectivity matrices can be used to construct brain networks from FC measures of the frequency domain (PLI, PLV, coherence), the source space domain, or the EC models (Sporns et al., 2004; Stam, 2004; Bullmore and Sporns, 2009). Subsequent connectivity metrics of all paired electrodes can then be explored between regions, using the Graph theory approach (Stam and Van Straaten, 2012). This method represents the brain as a collection of nodes, corresponding to recording sites or brain regions, and the pairwise relationship between them (edges). Taken together, nodes and edges enable the quantitative description of the local and global topological organization of brain networks (Van Diessen et al., 2015). It has been shown that small-world topology is found at different frequency bands (Stam, 2004) and can be associated with cognitive performance and developmental changes in functional brain networks in young children (Boersma et al., 2013).

Despite the growing number of published studies on language brain connectivity, the establishment of functional patterns of language networks during childhood and adolescence is not yet fully understood. In 2015, Weiss-Croft and Baldeweg (2015) published the first and only systematic review of studies that used fMRI to explore the development of functional language networks. The authors identified both progressive (increasing) changes of FC with age, associated with cerebral specialization, and regressive (decreasing) changes of FC with age, associated with more automatized language processing and lower engagement of control mechanisms (Weiss-Croft and Baldeweg, 2015). Specifically, their review highlights four main findings. First, brain activity in regions that support semantic processing increased throughout development, reflecting specialization of the brain. Second, with age, there is an increased activation in sensory–motor regions, along with a decreased activation in higher–order cognitive regions. Third, an age-related decreased activation was found in regions implicated in the default mode network (posterior cingulate cortex and precuneus). Finally, their results demonstrate the establishment of language lateralization by the age of 5 years. Although this study is indeed interesting, there is currently in the literature no systematic review that includes M/EEG studies. Because of the excellent temporal resolution of MEG and EEG, such a study would greatly help to provide additional and important information on the establishment of functional patterns of language networks. Therefore, the main objectives of this article are to provide a state of knowledge on the investigation of language-related cerebral networks in children, through the use of M/EEG, and a detailed portrait of relevant M/EEG data analyses methods that have been used in the assessment of language functional connectivity in children. To do so, we conducted this systematic review on functional, and to some extent effective, connectivity patterns of spoken language in children, as revealed by EEG or MEG. Given the multitude of metrics used to quantify oscillatory interactions (e.g., coherence, phase locking, connectivity matrices, graph theory, PSI) and the diversity of methodological designs (e.g., resting state vs. task recording, large variety of language tasks, longitudinal vs. cross-sectional study), the secondary objective is to synthesize and compare various method of connectivity analysis in the context of different pediatric populations (healthy and clinical) and a wide range of research objectives.

## Methods

### Search Strategy

The literature review was conducted using five databases: PubMed, PsycINFO, Web of Science, Scopus, and Linguistics and Language Behavior in order to find articles published between January 1995 and June 2018 inclusively. The key terms used were as follows: (magnetoencephalography OR electroencephalography OR MEG OR EEG) AND (resting state OR functional connectivity OR synchron* OR network* OR effective connectivity OR coherence) AND (Language OR Speech) AND (infant* OR infancy OR child OR children OR youth* OR toddler* OR schoolchild* OR teenager* OR adolescent* OR kid OR kids OR newborn). Additional reports were identified by handsearching the references cited in the retrieved articles.

### Selection Criteria

This review is limited to empirical studies published in peer-reviewed journals in English or in French. Studies that adhered to the following inclusion criteria were considered: (1) The study included children or adolescents (<18 years old), although the age range may extend into adulthood; (2) functional or EC analysis was performed based on EEG or MEG data. We verified whether the described methods allowed actual interpretation of functional connectivity or applied different techniques such as intertrial synchronization, ERP timing, or time-frequency analysis, which were sometimes referred to as functional connectivity, but do not in fact fall in this category (Sakkalis, 2011; Bastos and Schoffelen, 2016). (3) Studies that investigated language networks were included if either one of the following two conditions was met: (a) the authors used a behavioral assessment before or after the imaging acquisition, in order to evaluate language abilities; or (b) the authors applied expressive or receptive language paradigms (e.g., speech stimuli, story listening, or speech production) during MEG or EEG recording. In order to provide an exhaustive view of the connectivity patterns associated with language in childhood, this systematic review includes clinical pediatric samples as well as healthy children, as long as the methodology fit our selection criteria. Articles about written language only (reading or writing) without any association with verbal comprehension or expressions have been excluded.

**Figure 1:**
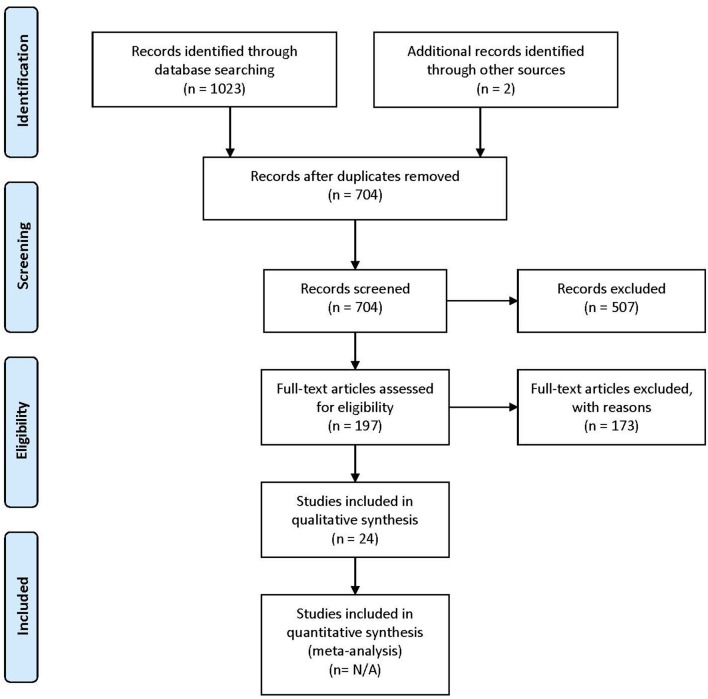
PRISMA (Preferred Reporting Items for Systematic Reviews and Meta-Analyses) flow diagram describing the paper selection process. Figure adapted from Moher et al. (2009).

The lists of references of the selected articles were searched manually for additional relevant articles. The study selection process is summarized in Figure 1.

### Data Extraction

Following the database search, duplicates were removed. For all remaining articles, titles and abstracts were reviewed by the first author (IG) and selected for a second revision if they met at least one of the inclusion criteria. For the second revision, remaining articles were reviewed independently by two authors (IG and AH), in order to determine whether they matched the purpose of this study. When no consensus was reached, the consultation of a third-party expert in the domain (PV) helped make the ultimate decision on eligibility. Figure 1 shows the PRISMA (Preferred Reporting Items for Systematic Reviews and Meta-Analyses) workflow diagram for study selection. Relevant information from each article was entered into a spreadsheet that included: (1) sample characteristics: age, gender, IQ, language evaluation method, sample size; (2) experimental paradigms: resting state, event-related experiments, sleep studies; (3) brain recording technique (EEG or MEG); (4) connectivity metrics.

The wide variability in study characteristics along these methodological dimensions precluded a meta-analysis. Instead, we synthesized and critically appraised findings made through the use of functional connectivity in the study of spoken language in children.

## Results

A total of 704 articles were screened in the first step. Of these, 507 were excluded on the basis of their title or abstract, either because they were not experimental studies (e.g., review), they were conducted with adult participants only, or they did not conduct connectivity analysis using EEG or MEG. Following these exclusions, 197 articles were assessed for eligibility. Of these, 173 were excluded because they did not meet at least one of the selection criteria.

A total of 24 articles met the selection criteria, passed interrater revision (79% agreement), and were confirmed by the third-party expert. All publications included in this work are peer-reviewed studies about FC of language functions in children, as revealed by EEG or MEG, and were published between 1999 and 2018. Detailed information was gathered about each study's population of interest, sample size, age of participants, design, imaging paradigm, type of language assessment, frequency bands considered for analyses, use of source or sensor analyses, and, finally, approach for connectivity analysis (see Table 1 for studies including healthy children and Table 2 for those addressing clinical populations). Each table begins with studies using EEG (Tables 1A, 2A) followed by those employing MEG (Tables 1B, 2B).

**Table 1A:** Descriptive data and methodological outline of articles focusing on healthy children in EEG studies.

**References**	***n* (M/F)**	**Age**	**Design**	**EEG/MEG paradigm**	**Language assessment**	**Frequency band(s)**	**Source/sensor**	**Connectivity analysis**
**EEG**								
Asano et al., 2015	13/6	11 mo	Cross-sectional	Symbol–sound mismatch	N/A	Alpha, beta	Sensor	Phase locking value
Hanlon et al., 1999	284/224	0–16.75 y	Cross-sectional	Resting	N/A	Theta	Sensor	Coherence
Kühn-Popp et al., 2016	15/17	14; 15 and 42 mo	Longitudinal	Resting	Declarative pointing and Verbal-IQ	Theta–alpha	Sensor	Coherence
Marshall et al., 2008	48/42	30 and 42 mo	Longitudinal	Resting	Reynell Developmental Language Scales	Theta, alpha, beta	Sensor	Coherence
Mundy et al., 2003	18/14	14–24 mo	Longitudinal	Resting	MCDI	Theta	Sensor	Coherence
Poblano et al., 2016	18/18	9–16 y	Cross-sectional	Resting; Lexical-tonal discrimination	N/A	Theta	Sensor	Pearson correlation
Whedon et al., 2016	153/147	6–34 mo	Longitudinal	Resting	PPVT-III^2^	Theta–alpha	Sensor	Coherence
Yang et al., 2005	23 (N/A)	6–8 y	Cross-sectional	Resting	Verbal-IQ	Delta, theta, alpha, beta	Sensor	Pearson correlation

**Table 1B:** Descriptive data and methodological outline of articles focusing on healthy children in MEG studies.

**References**	***n* (M/F)**	**Age**	**Design**	**EEG/MEG paradigm**	**Language assessment**	**Frequency band(s)**	**Source/sensor**	**Connectivity analysis**
**MEG**								
Doesburg et al., 2016	31/42	4–18 y	Cross-sectional	Word generation	PPVT, EVT	Alpha, beta, theta	Source	Phase locking value, phase lag index, graph theory
Doesburg et al., 2012	5/5	16–19 y	Cross-sectional	Word generation	N/A	Gamma, theta	Source	Phase locking value
Kadis et al., 2016	13/8	5–18 y	Retrospective	Word generation	N/A	All	Source	Phase slope index
Kikuchi et al., 2011	36/42	32–64 mo	Cross-sectional	Story listening	Expressive Vocabulary and Riddles (K-ABC)	Delta, theta, alpha, beta	Sensor	Coherence
Youssofzadeh et al., 2017	13/16	4–18 y	Cross-sectional	Word generation	N/A	Theta, alpha, beta, gamma	Source	Phase locking value

*Studies in the first part of the table used EEG, whereas those in the second part applied MEG*.

*M, male; F, female; N/A, not applicable; MCDI, Mac-Arthur communicative developmental inventory; PPVT, Peabody Picture Vocabulary Test; ECT, Expressive Vocabulary Test; K-ABC, Kaufman Assessment Battery*.

**Table 2A:** Descriptive data and methodological outline of articles focusing on children with or at risk of different clinical conditions in EEG studies.

**References**	**Pathology**	***n* (M/F)**	**Age**	**Design**	**EEG/MEG paradigm**	**Language assessment**	**Frequency band(s)**	**Source/sensor**	**Connectivity analysis**
**EEG**									
Righi et al., 2014	Risk of autism	54 (N/A)	6 and 12 mo	Longitudinal	Discrimination of consonants	Subtest of Mullen Scales of Early Learning	Gamma	Sensor	Coherence
Njiokiktjien et al., 2001	Nonverbal learning disorder/ Language disorder^1^	12/6 12/6	6–11 y	Cross-sectional	Resting	N/A	All	Sensor	Coherence
Zare et al., 2016	Risk of language disorder^1^	17/7	6 mo	Cross-sectional	Resting	N/A	Delta, theta, alpha1, alpha2	Sensor	Connectivity matrix, graph theory
Kabdebon et al., 2015	Prematurity/healthy	18/12 10/5	8 mo	Cross-sectional	Syllabic learning	N/A	Alpha, beta	Sensor	Coherence
Vasil'yeva and Shmalei, 2013	Stammering/healthy	47/0 59/0	3–5 y	Cross-sectional	Resting	N/A	All	Sensor	Coherence
Williams et al., 2012	Congenital heart disease	14/2	0–18 mo	Longitudinal	Resting	Bayley Scales of Infant Development	Beta	Sensor	Coherence

**Table 2B:** Descriptive data and methodological outline of articles focusing on children with or at risk of different clinical conditions in MEG studies.

**References**	**Pathology**	***n* (M/F)**	**Age**	**Design**	**EEG/MEG paradigm**	**Language assessment**	**Frequency band(s)**	**Source/sensor**	**Connectivity analysis**
**MEG**									
Kovelman et al., 2015	Autism/healthy	10 (N/A) 9 (N/A)	8–12 y	Cross-sectional	Discrimination of native and foreign language	N/A	All	Source	Coherence
Mamashli et al., 2017	Autism/healthy	29/0 17/0	9–15 y	Cross-sectional	Tonal discrimination	Social communication questionnaire	All	Source	Coherence
Molinaro et al., 2016	Dyslexia/healthy	9/11 10/10	8–14 y	Cross-sectional	Sentence listening	Verbal fluency, rapid automatized naming, pseudoword repetition, and phonemic deletion	Delta, theta	Sensor, Source	Coherence, partial direct coherence based on Granger causality
Lizarazu et al., 2015	Language disorder^a^/healthy	6/4 5/5	8–14 y	Cross-sectional	Listening of sounds	Reading of word and pseudoword lists, pseudoword repetition, and phonemic deletion	Delta, theta, beta, and gamma	Source	Phase locking value
Barnes-Davis et al., 2018	Extreme prematurity/term born	9/6 7/8	4–6 y	Cross-sectional	Story listening	PPVT, Expressive Vocabulary Test	Beta	Sensor	Phase slope and phase lag index

*Studies in the first part of the table used EEG, whereas those in the second part applied MEG*.

a *Language-based learning disorders (e.g., dyslexia, dysphasia)*.

*M, male; F, female; N/A, not applicable; PPVT, Peabody Picture Vocabulary Test*.

Thirteen of the articles covered in this review addressed functional connectivity and language functions in healthy children, whereas 11 included children at risk of or suffering from various clinical conditions. Table 3 shows the different populations included in these studies. The most studied pathologies were related to language impairments such as dyslexia, language learning disorders, and stuttering (20%), as well as autism spectrum disorder (ASD) (13%).

**Table 3:** Overall composition of samples included in all studies.

**Study population**	**% (*n*)**
Healthy	54 (13)
Autism spectrum disorder	13 (3)
Prematurity	9 (2)
Dyslexia	8 (2)
Language learning disorders	8 (2)
Stuttering	4 (1)
Congenital heart disease	4 (1)

**Figure 2:**
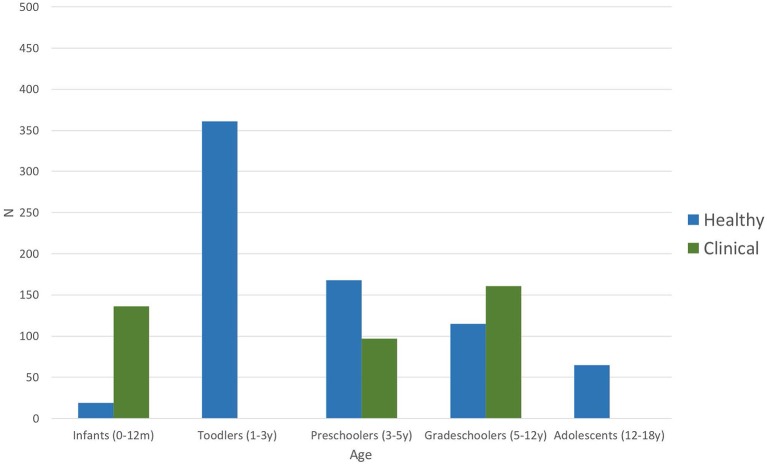
Number of participants per age group of all included studies (*n* = 24). Blue bars represent number of participants included in the articles addressing healthy children; green bars stand for the number of participants included in studies investigating clinical populations (including control groups) such as autism spectrum disorder, dyslexia, language-learning impairment, or prematurity (Table 3).

Figure 2 shows the distribution of the number of participants per age group taken together for all studies, both in healthy and clinical populations. Infancy includes the first year after birth (0–12 months). Toddlers are children aged between 1 and 3 years; preschoolers include the period from 3 to 5 years of age, grade-schoolers from 5 to 12 years, and adolescents are participants between 12 and 18 years of age. Each age group is subdivided into the number of children included in the studies addressing various clinical populations (green bars) and those interested in healthy children (blue bars), including those used as controls. Most of the healthy children studied were toddlers (*n* > 350), whereas studies interested in the impact of pathological conditions mostly included grade-schoolers (*n* > 150), although several studies on clinical populations also included infants and preschoolers. No data were available for any toddler or adolescent populations with clinical conditions. Overall, studies included in this systematic review total together a sample size of 728 in studies of healthy children and 394 in studies of clinical populations.

Different methods of connectivity analyses were used in these studies; they are summarized in Table 4. Some studies combined or compared several methods for estimating cerebral connectivity. Phase coherence analysis was the most common method used (45%), followed by PLV (21%). The analyses were based on all frequency bands, as specified in Tables 1, 2. The most studied frequency band was theta, and the least studied was gamma. Sixteen studies used sensor information, and seven applied a source analysis. One study reported results for both source and sensor-based analyses.

**Table 4:** Overview of all approaches applied to analyze functional or effective connectivity in included studies.

**Connectivity analysis**	% (*n*)^*^
Coherence	45 (13)
Phase locking value	21 (6)
Pearson correlation	7 (2)
Graph theory	7 (2)
Phase slope index	7 (2)
Phase lag index	7 (2)
Connectivity matrices	3 (1)
Granger causality	3 (1)

* *Some studies applied multiple analyses; hence the total n outranges the number of studies included in this review*.

Despite the fact that the scope of these studies differed, the aim of this review is to capture common findings concerning language-related functional connectivity. Therefore, we first present an overview of the results that emerged from the studies that investigated the association between language functioning and connectivity patterns, regardless of the task used during the EEG or MEG recording. Second, we illustrate, separately for healthy children and those in clinical populations FC and EC findings, while an expressive or receptive task was performed during the EEG and MEG recording. Finally, we display the results that emerge from all included studies organized according to the types of connectivity analyses used, beginning with those using functional connectivity, followed by those using EC. Again, the results will be indicated separately for healthy children and children with various clinical conditions.

### Overview of All Results

**Figure 3:**
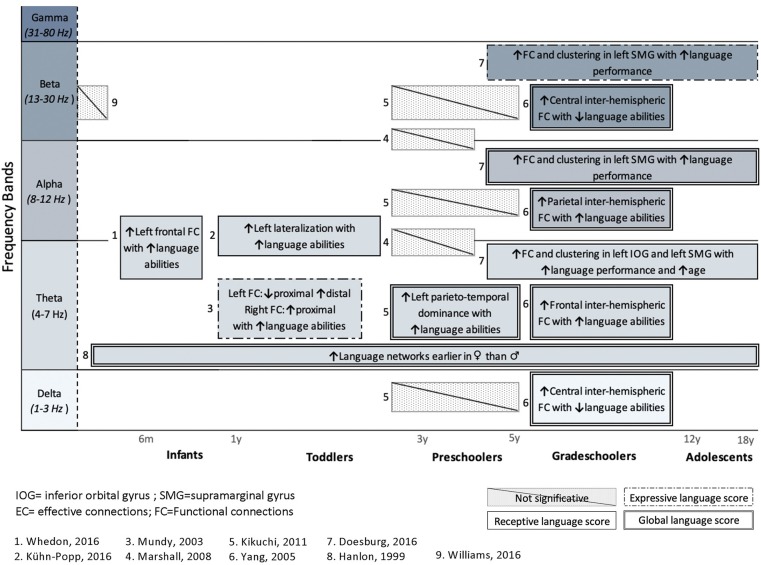
Summary of studies investigating the association between language abilities, assessed with standardized tools, and cerebral language networks. Results are presented for each frequency band and organized regarding ages. Studies in healthy subjects (*n* = 8) and a clinical population (n = 1) are included. Upper arrows (↑) indicate a positive correlation with either receptive (simple solid line), expressive (dashed lines 1), or global language functioning (solid double lines), whereas downward arrow (↓) indicates negative correlation with language. Hatched areas represent non-significant correlations with language abilities.

From the 24 articles included in the review, only nine attempted to associate FC or EC patterns with objective measures of language functioning. Figure 3 shows the main results from these nine studies, for healthy subjects (eight studies) and for a clinical population (one study). Results are presented for each frequency band and organized according to age.

Only one study (Williams et al., 2012) investigated the relationship between FC networks and language abilities in a clinical population, that is, children with congenital heart disease (CHD), who are known to be at high risk of language delay (Hövels-Gürich et al., 2008; Hövels-Gürich and Mccusker, 2016; Fourdain et al., 2019). The authors did not find any significant association between FC during the neonatal period and their later language abilities as measured at 18 months of age. Additionally, Marshall et al. (2008) found no significant correlation between FC patterns and language performance in preschoolers under foster care. However, seven studies found a significant relationship between FC in the theta band and language performance. Positive correlations between FC and language score were also found in higher frequency bands: alpha (Yang et al., 2005; Doesburg et al., 2016) and beta (Yang et al., 2005; Doesburg et al., 2016). It should be noted that no study investigated the relationship between language skills and FC patterns in the gamma band.

**Figure 4:**
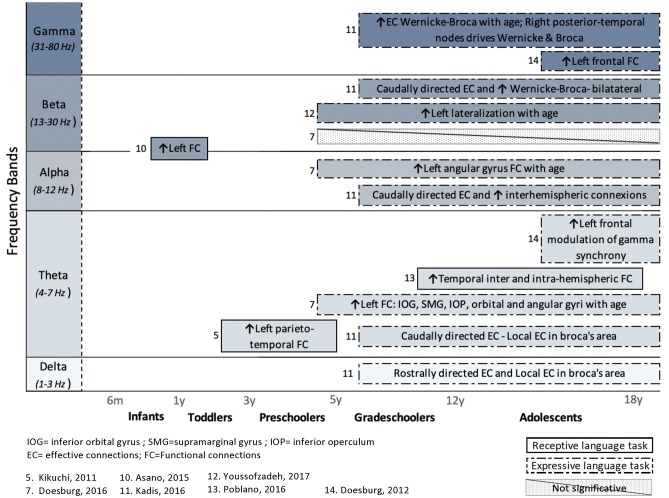
Overview of task-related connectivity patterns in healthy subjects. Results are organized regarding frequency bands and age groups investigated. Upwards arrows (↑) indicate an increased connectivity during receptive (simple solid line) or expressive (dashed lines) language task, whereas downwards arrows (↓) indicate decreased connectivity.

**Figure 5:**
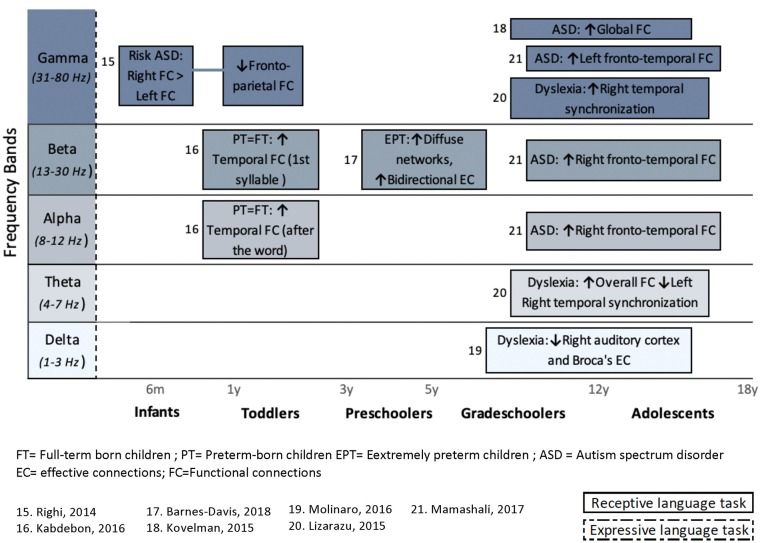
Overview of task-related connectivity patterns in clinical populations compared to healthy subjects. Upper arrows (↑) indicate an increased connectivity during either receptive (simple solid line) or expressive (dashed lines) language task in this clinical population compared to healthy children, whereas downward arrow (↓) indicates decrease FC correlation in this clinical population compared to healthy children.

In addition to articles that included a behavioral assessment of language functions, performed before or after an EEG or MEG recording, this systematic review also considers studies that included an expressive or receptive language paradigm (e.g., speech stimuli or speech production) during an MEG or EEG recording. The FC or EC patterns that arose from language paradigms are summarized in Figure 4 (for healthy children) and Figure 5 (for clinical populations).

In healthy children, the use of an expressive language paradigm (usually a verb generation task) was favored in four studies, whereas three studies used a receptive language task in order to examine the connectivity patterns that underlie language processing. These types of research paradigms have been performed mostly in research pertaining to grade-schoolers and adolescents, and the results are spread across all frequency bands.

In clinical populations, language tasks were mainly used to compare FC patterns between vulnerable children and healthy children. Here, only receptive language paradigms were used during M/EEG recording. Differences in FC between healthy and clinical subjects occur predominantly in the higher frequency bands (beta and gamma). Again, more details on the results of these studies are provided in section Results Derived From Connectivity Metrics.

Finally, it should be noted that two studies (Njiokiktjien et al., 2001; Vasil'yeva and Shmalei, 2013) done in resting-state FC in clinical populations were not presented in any of these figures. One of these studies looked at FC in children who received a diagnosis of language-based learning disorder (LLD), compared to children with non-verbal learning disorders (Njiokiktjien et al., 2001). The other looked at the FC patterns in children who stutter (Vasil'yeva and Shmalei, 2013). These studies did not use a language paradigm during EEG recording and therefore do not directly correlate connectivity patterns with behavioral language measures. The results of these two studies will nonetheless be discussed in section Results From Coherence in Clinical Population.

### Results Derived From Connectivity Metrics

#### Results From Correlation and Coherence Analyses

The correlation coefficient and its analog in the frequency domain, coherence, are the classic measures of interdependence between two signals (Sakkalis, 2011; Van Mierlo et al., 2014; Hassan and Wendling, 2018). Based on the amplitudes of the signals, the cross-correlation coefficient is a measure of the linear correlation between two time series and was utilized in one study using a tonal discrimination task (Poblano et al., 2016). Coherence, on the other hand, detects the linear relation between two electrophysiological signals at any particular frequency (Van Mierlo et al., 2014; Bowyer, 2016). It is mainly used at rest and appears to be the most popular metric for M/EEG evaluation of functional language networks in children (*n* = 13). One other study used coherence and Granger causality and will therefore be discussed in the section on EC.

##### Results from correlation in healthy children

In a study on adolescents (9–16 years old, Poblano et al., 2016), correlation analyses were performed between several recording sites of the brain and were acquired during a lexical-tonal discrimination task of bisyllabic words in the Zapotec language (a tonal language, spoken by the participants). Results showed significant increases of interhemispheric and intrahemispheric correlations of the theta-relative power during a word discrimination task, predominantly between left frontal and right temporal sites.

##### Results from coherence in healthy children

In healthy infants, few studies (*n* = 6) investigated the association between measures of coherence and later language abilities of preschoolers (Mundy et al., 2003; Marshall et al., 2008; Kikuchi et al., 2011; Kühn-Popp et al., 2016; Whedon et al., 2016) and grade-schoolers (Yang et al., 2005). Specifically, between 5 and 10 months of age, an increase in resting-state EEG coherence in the theta–alpha band (6–9 Hz) within left frontal regions seems to be associated with higher cognitive functioning, including receptive language at 3 years of age (Whedon et al., 2016). This association, however, might not be specific to language functions because the authors reported a mediating influence of the level of attentional control at the age of 2 years. Another study showed that, in the theta band (4–6 Hz), a pattern of less proximal (left-frontal to left-central) but more distal (left-frontal to left-occipital) resting state FC at 14 months old is negatively associated with the number of words expressed at the age of 2 years, as reported by the parents (lower vocabulary group; determined by the median split of the MacArthur Communicative Developmental Inventory (MCDI) results; Mundy et al., 2003). The same group also showed that at 18 months of age a ratio of higher proximal synchrony in the right hemisphere (right-frontal to right-central) is positively associated with vocabulary outcome (MCDI; total words) at 2 years old (Mundy et al., 2003).

At 14 months of age, a theta–alpha band (6–9 Hz), FC pattern of more proximal and less distal coherence appears to be specifically and positively associated with later language functioning, regardless of the child's IQ (Kühn-Popp et al., 2016). Accordingly, those results indicate that maturation of EEG coherence in the left hemisphere, established by the ratio of short-distance/long-distance connections, is positively correlated with preverbal communicative abilities at 15 months of age (e.g., pointing at objects) and with verbal communication skills at 48 months of age (epistemic language; Kühn-Popp et al., 2016). Congruently, left short-distance (parietotemporal) connectivity dominance in the theta band of preschoolers (32–64 months of age) during story listening shows exclusive positive correlation with language performance (no correlation with nonverbal cognitive performance or with chronological age), as assessed by the Kaufman Assessment Battery for Children at the same age (Expressive Vocabulary and Riddles subtests; Kikuchi et al., 2011).

In older children (6–8 years old), participants with high language functioning (verbal IQ >110, as assessed by the Wechsler Intelligence Scale for Children III) had an increased chance of higher correlations between homologous hemispheric regions (homologous interhemispheric correlations), compared to those who were classified as having a low verbal functioning (verbal IQ <90; Yang et al., 2005). This was apparent in several regions (frontal, parietal) and mostly in the theta and alpha bands. In contrast, higher connectivity in interhemispheric central regions (delta and beta) was associated with lower language abilities.

However, one study reported non-significant correlations between coherence indices and language functioning. Marshall et al. (2008) highlighted environmental impacts on cerebral connectivity in young children, even though no significant correlation with language or cognitive functioning was found. They reported that EEG patterns in 42-month-old children placed in foster care before the age of 24 months differed from those of children placed in institutional care, the former showing lower short-distance connectivity. Specifically, in the foster-care group, intrahemispheric connections between frontal-central and frontal-temporal regions were characterized by lower connectivity in theta–alpha (6–10 Hz) and alpha–beta (11–18 Hz) bands. The authors did not link this difference to language abilities (no significant results) but instead to environmental conditions (foster care vs. institutional care).

Finally, an extensive longitudinal study including 508 children between 2 months and 16.5 years of age investigated developmental differences between sexes, using EEG coherence (Hanlon et al., 1999). However, no behavioral data were used to associate coherence patterns with language functioning. Results illustrated a sex difference in development, whereby girls presented earlier development of comprehensive language networks in theta neural networks than boys. Results also suggested that girls have more complex interconnection patterns between paired sites, particularly in those involving the temporal lobes.

##### Results from coherence in clinical population

Coherence for FC analyses was also used in several studies that included children with or at risk of neurodevelopmental conditions and therefore known to have vulnerable language functions. More specifically, included in this section are those studies using coherence as FC analyses and that focused on children with ASD, CHD, language learning impairment (LLI), stuttering, and dyslexia.

Children with CHD are known to be at higher risk of speech and language delays (Hövels-Gürich et al., 2008; Hövels-Gürich and Mccusker, 2016; Fourdain et al., 2019) It is in this context that Williams et al. (2012) investigated the predictive value of neonatal EEG frequency power analysis for later language development in children with CHD. Results revealed predictive value of the delta-relative power for language skills at 18 months of age, as assessed by the Bayley Scales of Infant Development (BSID). However, association between language functioning and coherence measures did not achieve significant results, despite the high correlation between BSID cognitive scores and beta's interhemispheric (left frontal polar to right frontal polar) and intrahemispheric (left frontal polar to left occipital) coherence. According to the authors, this may have been due to the small sample size (*n* = 13 participants).

Autism spectrum disorder is a neurodevelopmental disorder commonly associated with verbal and communicative dysfunctions (Mcdaniel et al., 2018). In three studies identified in this review, alteration of language task-related coherence was associated with ASD (Righi et al., 2014; Kovelman et al., 2015; Mamashli et al., 2017). However, no direct association was made with language functioning.

One publication aimed to identify an early electrophysiological biomarker for later ASD diagnosis (Righi et al., 2014). Electroencephalography recordings were performed for 6-month-old infants at high risk (HR, meaning siblings of children that were already diagnosed with ASD) and low-risk (LR) for ASD, done while listening to speech sounds. A higher right than left hemispheric coherence in the gamma band was observed in all children, with no difference between groups (HR vs. LR). At 12 months of age, analyses in LR and HR groups revealed no remaining hemispheric lateralization differences. Interestingly, HR infants showed significantly reduced task-related FC between frontal and parietal regions, compared to LR infants. Although these results must be replicated using a larger sample, this association seems to identify a potential 12-month predictive marker for clinical outcomes (Righi et al., 2014). These results also point out that genetic vulnerability for autism, that is, having a full sibling diagnosed with ASD, can potentially be assessed in the first year of life, based on differences in neural integration.

The two other published studies that used coherence involved older children with confirmed ASD diagnosis. Important differences were identified in FC patterns between healthy children and those diagnosed with ASD. Results of a preliminary study by Kovelman et al. (2015) indicated differences in cerebral coherence between ASD and control groups (8–12 years old) during a language task. In particular, EEG coherence measures during familiarization with a new language, including statistical learning for discrimination between adjacent syllables, were higher in children with ASD and had predictive value for ASD diagnosis. Coherence measures during the familiarization phase showed improved identification of ASD diagnosis, compared to coherence measure at rest, thus suggesting that language learning abilities are different in children with ASD, compared to typically developing (TD) peers.

Finally, Mamashli et al. (2017) used an MEG tonal mismatch paradigm in children (9–15 years old) with ASD. The MEG recording revealed an increase in frontotemporal coherence in the ASD group relative to the TD group, in response to both standard and deviant stimuli. This manifested in the gamma band for the left hemisphere and in the alpha and beta bands for the right hemisphere. When coherence was normalized with respect to the standard condition, the differences between groups were no longer significant. However, when the same stimuli were presented against a noisy background, the normalized coherence remained greater in ASD group, and this for the beta band in the left frontotemporal regions (not illustrated in Figure 5). According to the authors, this may suggest that, for ASD children, reduced speech comprehension in noisy surroundings is due to a lower involvement of frontal control mechanisms. These results imply that auditory processing, when done against a noisy background, results in altered functional networks in this group of patients.

Overall, studies in children with ASD demonstrated several distinct characteristics of functional neuronal networks associated with auditory and language processing, which are in line with typical difficulties in language functions associated with ASD. Knowing the characteristics of cerebral networks could potentially allow an early identification of children at higher risk of developing ASD.

Two studies involved participants with oral language disabilities, such as language disorder or childhood-onset fluency disorder (stuttering). Vasil'yeva and Shmalei (2013) were interested in brain coherence of male preschoolers (3–5-year-old boys) with neurosis-like stammering. These children showed generally stronger global coherence in delta and beta oscillations than did healthy children. Compared to healthy controls, theta band synchrony in interhemispheric frontal regions was also increased for the stammering group, although a smaller number of connections was observed in children who stutter than in healthy children. Finally, in all frequency bands, interhemispheric coherence was higher in preschoolers with neurosis-like stammering than in the control group. These results suggest that, in children with this kind of speech disturbance, the specialization of functions of the left and right hemispheres, as well as the interhemispheric asymmetry, is less expressed.

Finally, for children (6–11 years old) with non-verbal learning disorders, Njiokiktjien et al. (2001) reported a right lateralized decrease of intrahemispheric coherence, in contrast with children with LLI, who showed reversed lateralization. This difference was higher in the gamma band. Again, these M/EEG FC results suggest that hemispheric functional brain alterations are related to specific language development disorders.

#### Results From Phase Synchronization

Instead of investigating the relation between the amplitudes of the signals, one could also evaluate how the phases of the considered signals are coupled, the so-called phase synchronization measures. Among the many phase synchronization measures proposed in the literature, one of the most used is the PLV, which evaluates the phase difference between two signals (Lachaux et al., 1999). When two brain areas are functionally connected, the phases of their signals are assumed to evolve together; therefore, the difference in their phases should be constant (Bruña et al., 2018).

##### Results from phase synchronization in healthy children

Three studies combined phase synchronization metrics: two with an FC matrix (Doesburg et al., 2012; Youssofzadeh et al., 2017) and one with EC metrics (Barnes-Davis et al., 2018). Results from these three will be included in the sections on graph theoretical approaches and EC, respectively.

Two other studies drew on phase synchronization metrics (PLV) in healthy children: one in a mismatch paradigm (receptive task) and the other in an expressive language task.

At around 1 year of age, results during an audiovisual paradigm revealed an increased large-scale communication between brain regions in the mismatch condition (a heard sound does not match the previously presented symbol), compared to the match condition (sound and symbol match; Asano et al., 2015). This occurred in the alpha–beta band (12–15 Hz) and was more prominent in the left hemisphere. According to the authors, this indicates that audiovisual integration requires a greater effort in the mismatch condition (Asano et al., 2015).

In adolescents (17 years old), an expressive language task (verb generation) resulted in an increased gamma-band synchronization among task-activated cortical regions (Doesburg et al., 2012). Moreover, there was a theta modulation of interregional gamma synchrony between several pairs of activated brain regions, mostly in the left frontal cortex. This reflects the involvement of gamma-band synchronization in language production and the role of low-frequency rhythms (theta), which modulate high-frequency connectivity in adolescents.

##### Results from phase synchronization in clinical population

One study used phase synchronization metrics (PLV) in task-related paradigms, in a vulnerable population, namely, children born prematurely. In fact, several studies report impairments of cognitive and behavioral functions, including language abilities, related to premature birth (weeks of gestation ≤37; e.g., Aarnoudse-Moens et al., 2009; de Kieviet et al., 2012). In our sample, one study used PLV for FC analyses in prematurely born children (27–30 weeks of gestation). Kabdebon et al. (2015) compared spatial synchrony and phase coincidence of EEG oscillations during syllabic learning in 8-month-old preterm-born and term-born children (corrected age for preterm-born). They did not find any differences between groups, suggesting similar language processing at 8 months of age. In both groups, an increase in the PLV was observed first in the beta band (13–18 Hz; during the first syllable) and later in alpha (8–12 Hz; after the word) over the left and right temporal areas (Kabdebon et al., 2015).

Using auditory stimuli in children (8–14 years old) and adults with dyslexia, another study found that, compared to a control group, dyslexic participants presented stronger synchronization and an absence of right hemispheric neural synchronization, related to low frequency (4 Hz; Lizarazu et al., 2015). On the other hand, for high frequencies (30 Hz), adults but mainly children with dyslexia show a rightward, instead of bilateral hemispheric lateralization. According to the authors, this may suggest that speech processing in dyslexic children relies more heavily on syllabic-rate information, compared to skilled reader peers.

#### Results From Network Analysis

Graph theory analysis looks at the brain as a complex network consisting of a collection of nodes connected by edges, in order to comprehend the topological organization of brain networks (Tahmasian et al., 2015).

##### Results from network analysis in healthy children

Two studies applied graph theoretical analysis into MEG results to investigate the organization of expressive language networks, from preschool age to adolescence (4–18 years old). Even though both used a verb generation task during MEG, and derived networks from phase synchronization metrics, their conclusions were not identical.

In the first of the two, results from a verb generation task revealed a developmental shift of the beta band lateralization in language production when children (4–6 years old) were compared to adolescents (16–18 years old): hubs were most lateralized in adolescents, whereas younger children showed a more bilateral distribution, or even a right-hemispheric pattern (Youssofzadeh et al., 2017).

The second study showed that connectivity within language-related areas (left angular gyrus, left precentral gyrus, right inferior orbital gyrus, and right rolandic operculum) increased with age (Doesburg et al., 2016). This was true for language production in the theta band. Increased FC during an expressive language task was also observed in higher frequency bands (alpha and beta). However, this increase was primarily found in brain areas associated with visual processing and thus might rather be associated with processing of the stimulus than to language-related task demands. Developmental analysis suggested significant differences between age groups: larger connectivity networks in adolescents (14–18 years old), compared to younger children (4–9 years old), and a stronger task-dependent increase of connectivity (expressed as theta coherence) in language-related areas, especially in frontal regions. Finally, theta-band connectivity measures showed a significant association with verbal language functioning (assessed with the Peabody Picture Vocabulary Test and the Expressive Vocabulary Test). Thus, the strength of task-dependent network connectivity was associated not only with a maturational pattern but also with language abilities (Doesburg et al., 2016).

##### Results from graph theoretical analysis in clinical population

Zare et al. (2016) developed a machine learning approach based on EEG network characteristics (efficiency and leaf number) in 6-month-old infants. They aimed at determining, based on family history, the risk of LLDs. Relying on functional connectivity measures, this work allowed for the accurate stratification of the children into low-risk (LR) and high-risk (HR) groups for LLD. Early brain networks revealed a reduced cortical communication capacity in HR infants, showing a network that was both decentralized (as revealed by the clustering index in the delta and alpha) and less efficient (as revealed by a decreased efficiency in the delta, theta, and alpha). Based on complex EEG patterns with support vector machine, it was possible to classify the children into HR and LR groups with approximately 80% accuracy (specificity of 89% and sensitivity of 92%).

#### Directionality of Language Networks (Effective Connectivity)

Effective connectivity reveals the directionality of information flow in particular brain regions and the causal and dynamic influences of one region on another (Stephan and Friston, 2010; Friston, 2011). Two methods of EC were used in the studies selected for review: partial directed coherence, a frequency-domain representation of the concept of Granger causality (Baccalá and Sameshima, 2001) and the PSI, a method based on phase differences in signals over a specified frequency range (Nolte et al., 2008).

##### Effective connectivity in healthy children

Only one study used EC metrics to study language networks in healthy children during an expressive language task. Kadis et al. (2016) reported an increased number of effective connections (PSI) with age, between 5 and 18 years. More importantly, different task-related EC patterns seemed to emerge among frequency bands. Analysis of lower frequency bands revealed more local, rostrally directed connectivity patterns in the left frontal region. At higher frequencies, EC increasingly involved distal and interhemispheric nodes. In alpha and gamma, bidirectional information transfer was observed between left and right frontal and posterior temporal nodes, whereas in the gamma band, the right posterior temporal region emerged as an important driver of Wernicke (left posterior temporal) and Broca (left frontal) regions.

##### Effective connectivity in clinical population

Phase slope index was also used to compare EC (PSI) and FC patterns (PLI) between extremely prematurely born children (EPT; <28 weeks of gestation) and their term-born (TB) peers [37–42 weeks of gestation; (Barnes-Davis et al., 2018)]. At preschool age (4–6 years old), bilateral functional networks, including temporal and parietal regions, were revealed in both EPT and TB children during a receptive language task. On the other hand, the beta band indicated increased FC in language networks, as well as a more diffused network in EPT children, compared to TB. Moreover, analysis of EC suggested more bidirectional connections in EPT within bitemporal areas of the network, compared to TB, where fewer bidirectional networks or more unidirectional networks were identified. Effective connectivity analysis also revealed that hyperconnectivity patterns in EPT were attributable to a greater information flux drive from the right hemisphere. Nevertheless, because those differences in connectivity patterns were not correlated with language performance, it was reported to be an effect of the clinical condition only (i.e., prematurity). Consequently, the authors assumed that their findings indicated an efficient reorganization of cerebral language networks, allowing the maintenance of language abilities in EPT children (Barnes-Davis et al., 2018).

Neuronal response while listening to low-frequency speech (<10 Hz), in grade-schoolers (8–14 years old) with dyslexia, was overall less synchronized, compared to normal readers (Molinaro et al., 2016). More specifically, during language stimulation (meaningful sentences), reduced delta synchronization and impaired feed forward functional coupling (partial directed coherence) were found between the right auditory cortex and the left inferior frontal gyrus.

## Discussion

We systematically reviewed 24 studies that assessed M/EEG functional networks associated with language in children. The great variability in study populations, sample size, and methodology precluded us from conducting a meta-analysis. Instead, we synthesized and critically appraised findings on the use of functional or EC in the study of spoken language in children.

### Summary of the Main Observations

In order to characterize functional networks involved in language development, first considered were results reported in 13 articles on the study of TD children, and which used FC and EC analyses. The findings of most of the reviewed studies suggested that theta neural oscillations play a crucial role in healthy language development. In the theta band, a greater left resting-state coherence in early childhood seems to be associated with higher language functioning, either at the time of M/EEG recording (Kikuchi et al., 2011) or at a later age (Mundy et al., 2003; Kühn-Popp et al., 2016; Whedon et al., 2016). In older children (grade-schoolers to adolescents), associations between connectivity patterns and language abilities are not found only in theta, but in most frequency bands (delta, theta, alpha, and beta). The differences in frequency bands in relation to age might reflect typical brain maturation. Indeed, cerebral maturation in children has been associated with a global decrease of slow-wave activity, including theta oscillations, and an increase in higher frequencies (Uhlhaas et al., 2010). Thus, even though theta-band connectivity shows significant correlation with language abilities at all ages (Figure 3), it is critical to look at all different frequency bands, especially in older children (grade-schoolers and adolescents).

Further, theta frequency band has been related to syllabic processing (Giraud and Poeppel, 2012; Meyer, 2018), and increases in theta activation have been found for tasks that include verbal working memory (Friederici and Singer, 2015; Meyer, 2018). Syllabic processing of human language constitutes one of the fundamental stages of bottom-up language processing, and there is evidence that it is established *in utero*, before term age (Mahmoudzadeh et al., 2013; Skeide and Friederici, 2016). The predictive value of theta coherence for early language comprehension in infants may thus be explained by the fundamental role of syllabic processing in later language acquisition. Given the assumed relation between theta band coherence and working memory, studies addressing language networks should also apply language paradigms that allow for the differentiation between higher-order cognitive functions and different stages of language processing.

The investigation of FC or EC networks using a language task during M/EEG recording reveals results distributed across all frequency bands. The involvement of the various frequency bands probably varies based on the nature of the task (e.g., active lexical discrimination vs. passive oddball paradigm), the language modality (expressive vs. receptive), and the level of language processing (e.g., syllabic vs. semantic). That being said, results from EC patterns in expressive language paradigm vary considerably depending on the frequency bands (Kadis et al., 2016). An age-related increase is shown in left effective connections, whereas higher frequencies reveal more bilateral effective connections with increasing age (Kadis et al., 2016).

For healthy children, the majority of studies using task-dependent connectivity analysis reveal increased left FC during receptive (Kikuchi et al., 2011; Asano et al., 2015) and expressive (Doesburg et al., 2012, 2016; Youssofzadeh et al., 2017) language paradigms. This occurs as early as 11 months of age (Asano et al., 2015) and appears to be constant throughout development. Interestingly, when it comes to examining the pattern of task-related FC in populations at risk of language disorders, in comparison with neurotypical children, differences are prominently characterized by a tendency for greater FC in the right hemisphere (Righi et al., 2014; Lizarazu et al., 2015; Mamashli et al., 2017).

Results from studies targeting clinical populations, mainly children at high risk of or suffering from language disabilities, also contribute to the understanding of the interactions between language abilities and the brain regions associated with language acquisition. In this review, we included 11 studies that addressed FC and EC patterns of language networks in different clinical populations. In children with speech disturbances (language learning disorders or stuttering), the functional specialization in the left and right hemispheres and the interhemispheric asymmetry typically seen in language networks seem altered (less hemispheric asymmetry observed). However, in populations at risk of language disabilities, such as ASD, preterm children, and infants with CHD, there are no clear or replicable FC profiles associated with language functioning that arise from the current literature. Although differences are observable between clinical and control groups, they seem to be more attributed to the signature of the underlying clinical condition, rather than to language functioning itself. More studies are needed to better understand the brain substrates of language alterations and vulnerabilities in these populations.

These results are consistent with the conclusion from Weiss-Croft and Baldeweg (2015), who found that left language lateralization was well established by the age of 5 years. However, our results suggest that, before the first birthday, left lateralization is already apparent when a receptive language paradigm is performed (Asano et al., 2015). Moreover, a greater left connectivity before 5 years of age has been correlated with better language abilities (Mundy et al., 2003; Kikuchi et al., 2011; Kühn-Popp et al., 2016; Whedon et al., 2016). Thus, M/EEG research points toward an earlier implementation of left lateralization in language networks than was concluded from studies done with fMRI. This is probably due to the suitability of electrophysiological techniques for studying young children. Furthermore, the impaired left lateralization in populations at risk of language impairments attests to the importance of the early development of left functional networks (Righi et al., 2014; Barnes-Davis et al., 2018) and its maintenance in later development (Lizarazu et al., 2015; Mamashli et al., 2017).

The developmental trajectory of FC of language networks evolves significantly with age, with the presence of greater connectivity networks in adolescents, compared to younger children (Doesburg et al., 2016; Kadis et al., 2016; Poblano et al., 2016; Youssofzadeh et al., 2017), but also more local and less bilateral networks as age increases (Kikuchi et al., 2011; Doesburg et al., 2016; Kadis et al., 2016). In line with findings of fMRI studies, strong local networks may actually reflect both processes related to cerebral specialization and automatized language processing, which require less top-down regulation and thus involves fewer network interactions (Weiss-Croft and Baldeweg, 2015).

Nonetheless, the exact timeline of maturational processes in language networks is not yet fully understood. This may be due in part to the great intervariability of typical development. Also, many studies included only a limited age range or did not have sufficient participants per age group to permit reliable conclusions regarding developmental changes. The importance of accounting for age-related changes has previously been emphasized in fMRI studies, in order to correctly interpret associations between network characteristics and language capacities (e.g., Weiss-Croft and Baldeweg, 2015; Rimmele et al., 2018). On the other hand, the methodological heterogeneity (e.g., language paradigms, cognitive assessments, connectivity algorithms) between developmental studies on brain correlates of language processing do not allow the drawing of a clear maturational timeline.

Finally, one should consider that sex differences may impact the development of FC patterns, as stated by Hanlon et al. (1999). In fact, the importance of integrating sex analysis in research is now well-established (Tannenbaum et al., 2019), and the sex differences of brain development have been documented (Gur and Gur, 2016, 2017; Kaczkurkin et al., 2019). In a recent systematic review, Etchell et al. (2018) highlighted sex differences in brain language structure and function. However, they concluded that these differences do not necessarily lead to differences in language task performance. It is therefore possible that boys and girls employ different but equally effective cognitive strategies for certain tasks, which leads to minor differences in performance as evidenced by brain function but not in the behavioral performance itself. Consequently, it is important that subsequent studies consider possible sex differences when characterizing language networks.

A better understanding of the association between language functions and the different characteristics of brain networks should include normal variation patterns that are not related to language difficulties. Understanding the normal development of functional language networks would enable earlier identification of children at risk of language difficulties. Currently, language impairment is often detected only at an age at which evidence of healthy language functions can be formally assessed (Prelock et al., 2008). When a pathology is present, however, it could be crucial to initiate early intervention in order to support language development and increase quality of life for these children.

### Methodological Considerations

This review shines light on the heterogeneity of methodological approaches used in the study of language functions in children, through the use of FC and EC. Beyond the neuroimaging method used (EEG vs. MEG), the type of analyses and their nomenclature vary greatly between research groups. Functional brain connectivity and EC analyses are indeed still recent, and to date, there is no consensus on which methods are to be advocated, highlighting the importance of summarizing the current state of knowledge and pursuing further research in this field. This would not only describe the various methods available, but also assess their respective pros and cons, in order to select the appropriate technique for specific experimental conditions and samples. This will ultimately support the production of more reliable and robust results and provide clear directions for future studies. Methodological heterogeneity is not only an issue in EEG and MEG, but also poses an obstacle to reliable conclusions about language networks estimated with other neuroimaging techniques, such as fMRI (Weiss-Croft and Baldeweg, 2015), hence the need to establish common standards of best practice.

Nevertheless, the number of M/EEG studies identified indicates that coherence and phase-locking measures may have high utility in language research, because these metrics were used in the majority of the published articles in the domain. These approaches achieved popularity because of their simple algorithms and fast computation. However, although coherence has been the most widely used FC method in this field, this does not necessarily mean it is the preferred method, nor the most fruitful. In fact, coherence may cause false-positive results, due to source leakage between local regions (Brookes et al., 2014; Kida et al., 2015). To overcome these challenges, many algorithms have been developed in the last few years. The Imaginary Part of Coherency (Nolte et al., 2008) and PLI are metrics that are less affected by the influence of common sources and active reference electrodes. They were introduced to facilitate the estimation of phase synchronization but have not been used much in the research of language development (none for Imaginary Part of Coherency and twice for PLI). Yet, the simplest method for reducing the influence of leakage on the estimation of connectivity is a leakage-invariant metric (O'reilly et al., 2017).

Conversely, the use of task-evoked EC metrics such as Granger causality and PLI in this context is recent and remains limited, given that only three research teams have applied them since 2016. Thus, little is known about the directionality (EC) of oral language networks in children.

To date, the use of EEG is more frequent than MEG for the investigation of language-related brain connectivity in children (14 and 10 articles, respectively), certainly because of the higher accessibility, lower cost, and ease of use of the EEG technique.

#### Methodological Limitations of Reviewed Studies

The primary methodological limitation of most studies reviewed was the failure to directly examine the association between brain FC patterns and objective language skills as assessed by standardized behavioral tests. In addition, in those studies that did evaluate language abilities, assessment of overall cognitive functioning was not always performed. Thus, the observed disturbance could indicate a lower global cognitive functioning, rather than a specific effect of language difficulties. A clear distinction between language and global cognitive functioning is therefore critical when investigating links between connectivity patterns and language performance. Relationships between brain activity and behavior must be addressed, especially in the context of clinical populations, where the disturbance in FC patterns associated with the neurodevelopmental condition must be distinguished from the disturbance specific to language functions alterations. For instance, in contrast to healthy children, M/EEG FC differences in children with CHD or born prematurely are not always associated with actual differences in language skills. The lack of attention to these relationships may be partially explained by the small sample sizes of the studies, which led to poor statistical power.

Finally, the results from various studies emphasized the difficulty of applying FC analysis derived from M/EEG data. Source localization of cerebral activity, captured on the surface of the scalp, represents a particular challenge for sensor-space analysis. This is known as the inverse problem, which may lead to inaccurate identification of cerebral networks (e.g., Nunez et al., 1997; Sakkalis, 2011; Barzegaran and Knyazeva, 2017; Abreu et al., 2018, 2019). Also, the effect of volume conduction, which is a mix of several signals within one sensor, and which originate from identical cerebral regions, makes critical a direct derivative from sensors to cerebral representation. Source-space analysis tries to overcome this downside and uses models that aim for a more accurate reconstruction of the true sources of the signal (Schoffelen and Gross, 2009). The conduction of source analyses seems particularly important when one is aiming to interpret FC, because the same cerebral activation is measured with different sensors and may potentially result in false conclusions regarding connected regions. Recently, it has been shown that source-space analyses seem accurate mostly when using high-density EEG, but result in limited interpretation of the more common low-density EEG (Barzegaran and Knyazeva, 2017). Also, some of the approaches to source analysis require certain assumptions be made about the underlying network, which may not be accurate for all data sets (Daunizeau and Friston, 2007). In particular, in children (where networks are developing) or in clinical populations (where networks may be altered), it can be risky to assume a certain network composition. These limitations need to be taken into consideration when interpreting some of the findings on functional networks that are reported in this review. While studies that applied sensor-space analysis may overestimate functional connectivity, the interpretation of findings based on source-space analysis, especially in low-density EEG, may be less susceptible to this same overestimation. Finally, some studies might not have verified specific assumptions for their source-model, which limits their interpretation. This issue may occur especially in studies that include clinical populations, where characteristics of cerebral activation may be altered.

#### General Utility of M/EEG Connectivity Analysis

By providing information about temporal coupling between cortical areas (milliseconds time scale) and frequency bands of neural oscillations, both MEG and EEG are well-suited to study the development of language networks. They offer a quiet testing environment, which facilitates the use of language tasks. Moreover, they provide excellent temporal resolution, allowing analyses that target an immediate response to specific tasks or stimuli.

Because EEG is less sensitive to movement than other techniques (e.g., fMRI), thus allowing a certain mobility and tolerating articulatory movements, it is highly relevant for language assessment in pediatric populations. Furthermore, the low cost of EEG justifies its use for the investigation of developmental trajectories, which requires longitudinal design with multiple recordings over time. On the other hand, spatial and temporal data available from MEG allow the investigator to track both the neural timing and location associated with language and thus to efficiently map the trajectories of language networks. Regardless of the neuroimaging technique employed, the use of FC is highly relevant in research on children, because it allows acquisition at rest, without requiring that a task be performed, as it is in traditional ERP paradigms. Furthermore, the length of time required for data acquisition can usually be shorter, compared to task paradigms. Finally, a better understanding of FC M/EEG analysis and an evaluation of their usefulness are essential for future research and for the potential use of these techniques in clinical contexts.

### Limits of This Review

Although this systematic review goes beyond a simple revision of the literature, it does not include any statistical analysis of the reviewed studies, as would have provided a meta-analysis. The reader should therefore take into account the fact that the current findings represent qualitative and not quantitative results. The methodological heterogeneity of the included studies, with respect to their paradigms, the types of FC and EC analysis, as well as the large age range of the children investigated, is in itself a limitation for the generalization and integration of the results.

Compared to other neuroimaging techniques, both MEG and EEG stand out because of their high temporal resolution. This is of particular importance in language paradigms, where tonal differences occur at a fast rate. However, both methods have a relatively low spatial resolution, which leads to a rather large-scale localization of cerebral activity when compared to techniques such as fMRI. Thus, the present findings about functional language brain networks permit only limited spatial interpretation.

Finally, given that we mainly reviewed studies that considered FC as a measure of neuronal networks, we would like to acknowledge that FC bears an index of statistical dependency. More precisely, it allows the estimation of the correlation between cerebral activation, measured simultaneously with different electrodes or sensors located over different cerebral locations. Thus, it does not allow causal conclusions about brain networks. Only three studies (Kadis et al., 2016; Molinaro et al., 2016; Barnes-Davis et al., 2018) included EC analysis that allowed causal conclusions about interactions within functional language networks. Future studies should definitely include EC analysis that allows for more advanced characterization of cerebral language networks.

## Conclusion and Future Directions

The analysis of brain functional connectivity and EC through the use of M/EEG data is a common emphasis of ongoing developmental research, but many unanswered questions remain regarding the brain correlates of language development. To our knowledge, this is the first systematic review to summarize the current state of knowledge on linguistic electrophysiological patterns of brain connectivity in the pediatric population. It provides a detailed portrait of the relevant MEG and EEG data analysis methods that have been used in that context. Future research should consider the different FC analyses available, in order to choose the appropriate tools and paradigms. Overall, the results of the reviewed studies are highly heterogeneous, precluding the possibility of drawing clear and quantitative conclusions and showing the importance of pursuing research in this field. Future work will enlighten on the brain substrates of language development and may also have important clinical impacts, for example, leading to the identification of early neuroimaging markers associated with altered language development in populations at high risk of language disabilities. It would also allow the identification of children at higher risk of language difficulties, in order to provide early and individualized intervention (Jeste et al., 2015). However, studies with significantly larger sample sizes, as well-normative data, are needed in order to be able to use these tools in a clinical context.

## Data Availability Statement

Datasets are available upon request to the corresponding author.

## Author Contributions

IG developed search procedure, performed database searches and reviewed articles for inclusion/exclusion based on the title, and abstract. IG and AH reviewed articles for inclusion/exclusion based on the full text, extracted data from articles, analyzed the extracted data, and wrote the manuscript. AG supervised all aspects of the systematic literature review, preparation of the manuscript, revision, editing, and final intellectual content. PV contributed to intellectual content and provided comments on the manuscript.

### Conflict of Interest

The authors declare that the research was conducted in the absence of any commercial or financial relationships that could be construed as a potential conflict of interest.
